# De Novo Transcriptome Assembly and Characterization of the Synthesis Genes of Bioactive Constituents in *Abelmoschus esculentus* (L.) Moench

**DOI:** 10.3390/genes9030130

**Published:** 2018-02-27

**Authors:** Chenghao Zhang, Wenqi Dong, Wei Gen, Baoyu Xu, Chenjia Shen, Chenliang Yu

**Affiliations:** 1 Vegetable Research Institute, Zhejiang Academy of Agricultural Sciences, Hangzhou 310021, China; zhchhao2008@163.com (C.Z.); dwq9516@sina.com (W.D.); gengwei@mail.zaas.ac.cn (W.G.); sheshizaipei@163.com (B.X.); 2 Key Labortatory of Creative Agricultrue, Ministry of Agriculture, Zhejiang Academy of Agricultural Sciences, Hangzhou 310021, China; 3 College of Life and Environmental Science, Hangzhou Normal University, Hangzhou 310036, China; shencj@hznu.edu.cn

**Keywords:** flavonoids, glycosyltransferase, polysaccharide, okra, transcriptome

## Abstract

*Abelmoschus esculentus* (okra or lady’s fingers) is a vegetable with high nutritional value, as well as having certain medicinal effects. It is widely used as food, in the food industry, and in herbal medicinal products, but also as an ornamental, in animal feed, and in other commercial sectors. Okra is rich in bioactive compounds, such as flavonoids, polysaccharides, polyphenols, caffeine, and pectin. In the present study, the concentrations of total flavonoids and polysaccharides in five organs of okra were determined and compared. Transcriptome sequencing was used to explore the biosynthesis pathways associated with the active constituents in okra. Transcriptome sequencing of five organs (roots, stem, leaves, flowers, and fruits) of okra enabled us to obtain 293,971 unigenes, of which 232,490 were annotated. Unigenes related to the enzymes involved in the flavonoid biosynthetic pathway or in fructose and mannose metabolism were identified, based on Kyoto Encyclopedia of Genes and Genomes (KEGG) pathway analysis. All of the transcriptional datasets were uploaded to Sequence Read Archive (SRA). In summary, our comprehensive analysis provides important information at the molecular level about the flavonoid and polysaccharide biosynthesis pathways in okra.

## 1. Introduction

Okra (lady’s finger; *Abelmoschus esculentus* (L.) Moench), a crop which is a member of the mallow family, is widely distributed in tropical to subtropical regions [1]. Okra is grown as a health protective vegetable in China and it has a great deal of medicinal values. Several bioactive constituents of okra, including flavones, polysaccharides, pectin, alkaloids, and linoleic acid, were identified in previous reports [2,3,4]. These bioactives can help digestion, and have anti-cancer, anti-tumor, anti-diabetes, antibiosis, anti-inflammatory properties, confer liver protection, and other health care effects [5,6,7]. 

Previous studies centered on the purification and characterization of bioactive substances in okra. Water extract of okra pods are very thick, due to the high concentration of polysaccharide-based mucilages, which are frequently used as thickening agents for soups [8]. Okra polysaccharides can also be used instead of egg white as a fat substitute for chocolate biscuits and chocolate frozen desserts [9,10]. Of polysaccharides from okra mucilage reacted with a number of plant-based agglutinins, with the greatest response to mistletoe toxic lectin-I (ML-I), as well as precipitating well with agglutinins from *Abrus precatorius*, *Momordica charantia*, and *Ricinus communis* [11]. It was found that okra polysaccharide was composed of rhamnose, galactose, and galacturonic acid, and also contained rhamnogalacturonan I, which is an acidic polysaccharide [12,13]. The anti-tumor and immune-stimulatory activities of natural polysaccharides have attracted much attention, due to their low toxicity and minimal side effects [14,15,16]. OFPS11, a water-soluble polysaccharide, has been extracted and purified from okra flowers [17]. Three purified fractions of okra polysaccharides were obtained by water extraction and alcohol precipitation [18]. A new flavonol glycoside (5,7,3′,4′-tetrahydroxy-4′′-*O*-methyl flavonol -3-*O*-β-D- glucopyranoside) was isolated from the fruit of okra [19]. Reverse-phase high performance liquid chromatography (RPHPLC) was used to determine the content of the three flavonoid glycosides and the total flavonoid in different species, as well as in different tissues and organs of okra [20]. High performance liquid chromatography (HPLC) method was used to determine the contents of the five flavonoids (rutin, hyperoside, isoquercitrin, myricetin, quercitrin) simultaneously in the *Abelmoschus moschatus Medicus* capsule [21]. 

Previous studies on okra have mainly focused on genetic breeding, cultivation techniques, nutritional components, and medicinal efficacy [22,23,24,25,26]. Most of the molecular biology studies on okra were based on molecular markers, such as inter-simple sequence repeat (ISSR) and sequence-related amplified polymorphism (SRAP) markers, to analyze the genetic diversity of okra germplasm resources [27,28]. Because the okra genome has not been sequenced, there is little published research on the identification and organ-specific expression patterns of the synthesis genes associated with the synthesis of bioactive constituents in this crop. In the current study, transcription databases from the roots, stems, leaves, flowers, and fruits of okra were established. Very large numbers of unigenes related to flavonoid and polysaccharide biosynthesis were identified. Our transcriptome data are a useful resource to investigate expression profiles of the biosynthesis genes of bioactive constituents in okra.

## 2. Materials and Methods 

### 2.1. Plant Materials

Okra (*Abelmoschus esculentus* cv. Xianzhi) plants were grown in the farm of Zhejiang Academy of Agricultural Sciences in the Zhejiang Province of China under natural conditions. Roots, stems, and leaves were collected from four-week-old okra seedlings. Flowers were collected after opening; fruits were harvested at 5 days after anthesis. Three biological replicates were taken for each organ. Samples were immediately snap-frozen in liquid N_2_, and then stored at −80 °C until they were used. 

### 2.2. RNA Isolation and Library Construction

Each sample from five organs was produced as an independent pool (a total of 15 pools). Total RNA was extracted using the RNAiso for Polysaccharide-rich Plant Tissue reagent (TAKARA, Dalian, China) according to the manufacturer’s protocol, and was then quantified using a Nanodrop8000 (ThermoFisher, Waltham, MA, USA) and Agilent 2100 Bioanalyzer (Agilent, Santa Clara, CA, USA) with RIN number >7.0. The sequencing libraries were generated using the VAHTS mRNA-Seq v2 Library Prep Kit for Illumina^®^ (Vazyme, Nanjing, China) following manufacturer’s recommendations. Firstly, mRNA was purified from total RNA using poly-T oligo-attached magnetic beads. Fragmentation was performed using divalent cations under elevated temperature in Vazyme Frag/Prime Buffer (Vazyme). The cleaved RNA fragments were copied into first strand cDNA using reverse transcriptase and random primers. Second strand cDNA synthesis was subsequently performed using buffer, dNTPs, DNA polymerase I, and RNase H. Then, the cDNA fragments were end repaired with the addition of a single “A” base at the 3′-end of each strand, and subsequently ligated with the special sequencing adapters (Vazyme). The products were purified, and size selected with VAHTS^TM^ DNA Clean Beads (Vazyme) in order to get appropriate size for sequencing. PCR was performed, and aimed products were purified finally. PCR was performed with Phusion^®^ High-Fidelity DNA Polymerase (New England Biolabs, Ipswich, MA, USA). The number of cycles was adjusted according to the content of cDNA. For example, the amount of cDNA is 1 μg, using 6 cycles; when the amount of cDNA is 50 ng, with 10 cycles. Agilent 2100 Bioanalyzer (Agilent) system was used for library quality control.

### 2.3. Sequencing, Quality Control, and De Novo Assembly 

The raw reads were obtained from an Illumina HiSeq X Ten platform (Illumina, San Diego, CA, USA), and then filtered to exclude reads containing adaptors, reads with >5% unknown nucleotides, and low-quality reads with >20% of bases with a quality value ≤10. The obtained clean data was used to calculate the Q20 (proportion of nucleotides with quality value larger than 20), Q30 (proportion of nucleotides with quality value larger than 20), and GC-content. A de novo strategy was employed to assemble the transcriptome of okra based on a total of 104.375 Gb of data, using the Trinity assembly program with default parameters to form contigs [29]. TIGR Gene Indices clustering tool (Tgicl) was used to cluster assembly sequences and redundancy removal (setting: -l 40 -c10 -v 25 -O′-repeat_stringency 0.95 -minmatch 35 -minscore 35′) [30]. The assembly sequences no shorter than 200 were abstracted as unigenes for subsequent analysis. All of the transcriptional data were released in Sequence Read Archive (SRA) (SRP130180).

To identify transcripts homologous to those in model species, the functions of unigenes were annotated by BLAST program (*e*-value 1 × 10^−5^) against protein databases, including NCBI (National Center for Biotechnology Information) non-redundant protein sequences (Nr), Swiss-Prot protein, Gene Ontology (GO), Clusters of Orthologous Groups of proteins (COG) and Kyoto Encyclopedia of Genes and Genomes (KEGG) databases. Annotation with the associated metabolic pathway was achieved by KEGG, and GO annotation was carried out by Blast2GO [31]. Gene ontology functions of all unigenes were classified by the Web Gene Ontology Annotation Plot (WEGO) program [32].

### 2.4. Calculation of the Expression Level of the Transcripts and Analysis of Differentially Expressed Genes

The technique of fragments per kilobase of transcripts per million mapped reads (FPKM) was used to calculate the expression level of each unigene. Differentially expressed unigenes were analyzed using DESeq2 package, based on the negative binomial distribution test [33]. The thresholds of significant differentially expressed unigenes in comparisons between any two organs were FDR (False discovery rate) ≤ 0.05 and |log2Fold change| ≥ 1. MultiExperiment Viewer [34] (MeV) (version 4.9.0) was used to group all differentially expressed genes (DEGs) into 25 clusters by a K-means algorithm, based on their log_2_ values of transcript abundance [35]. 

### 2.5. Determination of Total Flavonoids and Polysaccharides in Okra

Samples of the five organs were collected as described in Section 2.1, with three replicates. To determine the polysaccharide contents in each of five different organs, phenol–sulfuric acid method was applied in this study. Briefly, the dried samples were ground into fine particles. An aliquot (0.1 g) of powdered sample was defatted with 80% (v/v) ethanol under continuous stirring at 90 °C for 3 h, and then filtered, and the sample washed with 80% (v/v) ethanol three times. The dried residue was extracted with 150 mL of double-distilled water for 3 h at 90 °C. The residue was washed three times with double-distilled water. Each sample produced 200 mL of combined aqueous extracts. An aliquot (1 mL) of the aqueous extracts was added to 1 mL phenol solution and 5 mL concentrated sulfuric acid solution and incubated for 30 min in a water bath at 40 °C. After cooling, the absorbance of 490 nm was determined to quantify polysaccharide concentration. To determine the concentration of total flavonoids in each plant organ, the Plant Flavonoids test kit (Jiancheng Bioengineering, Nanjing, China) was used, according to the manufacturer’s instruction.

## 3. Results 

### 3.1. Results of De Novo Assembly and Unigene Annotation

Three replicate samples from each of the five organs, namely roots, stems, leaves, flowers, and fruits, were collected from okra for paired-end transcriptome sequencing. A total of 716,330,252 raw reads were obtained from all the cDNA libraries. After filtering out adaptors and low-quality reads, 695,836,110 clean reads were generated, containing 104.375 Gbp clean data (Table 1). The number of clean reads in the roots was 46,916,458 (97.22% of the raw reads), 47,097,672 (97.27% of the raw reads) and 46,112,238 (97.24% of the raw reads) clean reads for the three replicates. For stems, the values were 47,316,422 (97.26%), 47,323,464 (97.19%), and 43,132,910 (97.242%) clean reads. For leaves, the values were 47,341,802 (97.36%), 46,142,720 (97.30%), and 49,272,238 (97.34%) clean reads. For flowers, the values were 42,777,662 (97.05%), 45,883,906 (97.10%), and 41,960,012 (96.87%) clean reads. For fruits, the values were 49,905,802 (96.93%), 48,083,880 (97.26%), and 46,568,924 (97.25%) clean reads. Trinity software was used to assemble the clean reads of each sample. Finally, a total length of 0.38 Gb unigenes was generated, with the mean length of a unigene being 1297 bp (N50: 1885 bp) (N50 value defined as the length for which the collection of all sequences of that length or longer contains at least half of the sum of the lengths of all sequences) (Table S1). 

**Figure 1:**
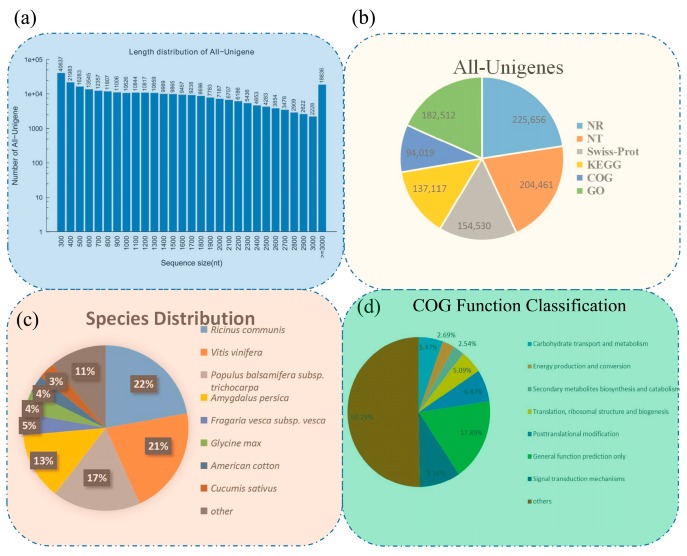
Characteristics of unigenes generated by Illumina sequencing. (**a**) The length distribution of all assembled unigenes. (**b**) The number of unigenes annotated by different databases, including NR, NT, Swiss-Prot, KEGG, COG, and GO. (**c**) Species distribution of the top BLAST hits for all homologous sequences. (**d**) COG function classification of all unigenes. NR: Non-redundant; NT: Nucleotide; KEGG: Kyoto Encyclopedia of Genes and Genomes; COG: Clusters of Orthologous Groups; GO: Gene Ontology.

The distribution of unigene lengths are shown in Figure 1a, and a summary of the sequencing output results and assembly quality statistics are listed in Table 1 and Table S1, respectively. To determine functions of the assembled unigenes in okra, all of the unigene sequences were run against six databases, using BLASTX (with *e*-value as 1 × 10^−5^) (BLASTN for Nt database (NCBI non-redundant nucleotide sequences), with *e*-value as 1 × 10^−10^). In total, 225,656 unigenes are annotated in the Nr database, 204,461 in the Nt database, 154,530 in the SwissProt database, 137,117 in the KEGG database, 94,019 in the COG database, and 182,512 in the GO database (Figure 1b). Based on these annotations, 232,490 of the okra unigenes have at least one hit in six databases. As the genome of okra has not been published, we blasted the sequences of all unigenes against other species via the Nr database. A total of 783 plant species exhibited mRNA sequences homologous to okra unigenes. The results showed similarity overlap between okra and *Ricinus communis* (21.55%), *Vitis vinifera* (20.58%), *Populus* *trichocarpa* (16.78%), *Amygdalus persica* (12.85%), *Fragaria vesca* ssp. *vesca* (4.2%), *Glycine max* (4.2%), and *Gossypium hirsutum* (3.31%) (Figure 1c).

### 3.2. Classification of Enriched Gene Ontology Terms and KEGG Pathways

**Figure 2:**
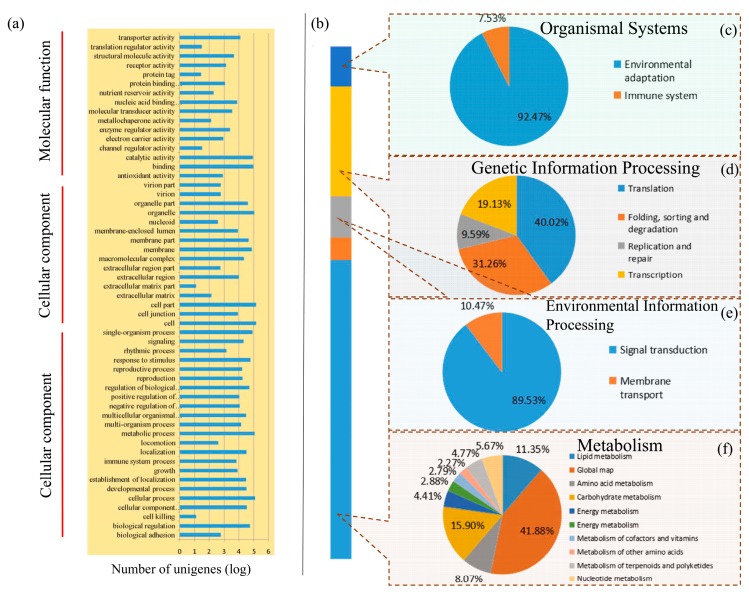
Gene Ontology and KEGG terms assignment of okra unigenes. (**a**) A total of 182,513 unigenes were assigned to different GO terms. (**b**) A total of 137,117 unigenes were assigned to different KEGG terms. Different color blocks represent different terms, from top to down, “Organismal Systems”, “Genetic Information Processing”, “Environmental Information Processing”, “Cellular Processes” and “Metabolism”. Proportion of unigenes in each second level term in “Organismal Systems” (**c**), “Genetic Information Processing” (**d**), “Environmental Information Processing” (**e**), and “Metabolism” (**f**).

The GO database was used to categorize standardized gene functions. GO analysis organized the predicted functions of unigenes into three main ontology terms: molecular function, cellular component, and biological process. Based on the information from the Nr annotation, we obtained the GO function annotation by Blast2GO and WEGO software (Figure 2a) [32]. In the biological process GO term, the most frequent was “cellular process”, with 122,914 unigenes. In the cellular component ontology, “cell” and “cell part” were the most highly represented groups, with 140,964 and 140,713 unigenes, respectively. Lastly, in the molecular function category, the largest numbers were found in “binding” (92,021) in molecular function category. Furthermore, we compared all unigenes against the COG database to predict the possible functions of these unigenes. A total of 187,006 unigenes were matched and allocated to 25 functional categories (Figure 1d). The three most frequent categories were “General function prediction only” (33,456 unigenes, 17.89% of the total), “Transcription” (19,635, 10.50%), and “Signal transduction mechanisms” (17,134, 9.16%). The COG analysis helped us to understand the protein function distribution characteristics and functional classification statistics in okra.

To identify the biological functions of the okra genes, all of the assembled unigenes were classified into KEGG pathways by using BLASTX against the KEGG database. A total of 137,117 unigenes were classified into five main KEGG functional subcategories, including organismal systems, genetic information processing, cellular process, environmental information processing, and metabolism (Figure 2b). In total, the unigenes were assigned to 128 KEGG pathways. The most heavily enriched KEGG pathways were related to metabolic pathways. In “genetic information processing” subcategory, the “translation” term contained the largest number of unigenes (40.02%) (Figure 2d). In the “metabolism” subcategory, the “Global map” term contained the largest number of unigenes (41.88%) (Figure 2f).

### 3.3. Differential Gene Expression Analysis and KEGG Pathway Classification among Different Organs

**Figure 3:**
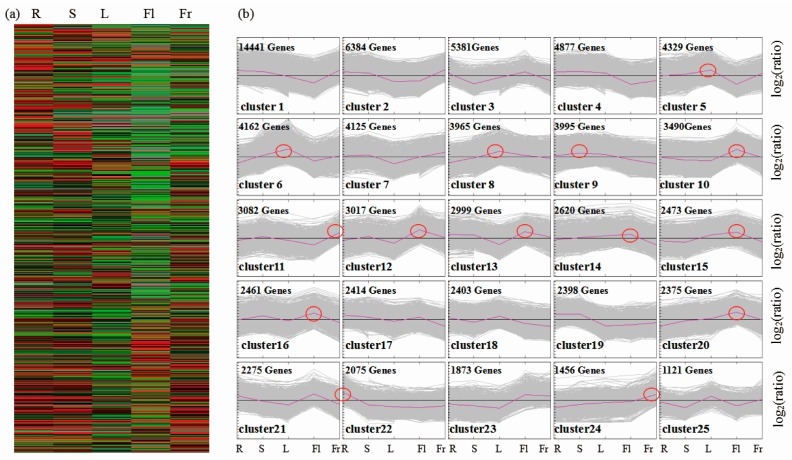
Transcriptional variation between five different organs of okra. (**a**) Expression profiles of the differentially expressed unigenes (DEGs) between five different organs are illustrated by a heatmap. (**b**) Cluster analysis by the K-means method from the gene expression profiles. Red circles indicated the organ-specific expressed genes.

To identify genes which were differentially expressed among the five organs, the FPKM values were used. Based on our data, and analyzed using a significance threshold of at least a 2-fold difference in expression and a FDR < 0.05, a total of 90,601 significant DEGs were identified between the fruits and any of the other four organs (Figure 3a). To show the major trends in organ-specific expression between five organs, all 90,601 differentially expressed unigenes were assigned to 25 clusters by MeV software, using hierarchical clustering (HCL) and the K-means clustering method. Transcript levels of unigenes in cluster 22 were higher in roots than in the other organs; unigenes in cluster 9 were highly expressed in stems, while unigenes belonging to clusters 5, 6, and 8 were highly expressed in leaves. Unigenes in clusters 10, 12, 13, 14, 15, 16, and 20 were highly expressed in flowers, while transcript levels of unigenes in cluster 11 and 24 were very high in fruits (Figure 3b).

**Figure 4:**
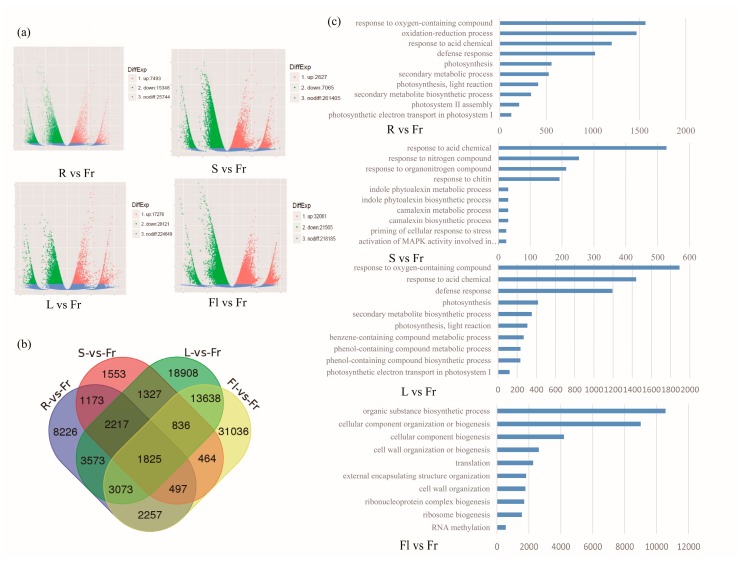
Overview of the DEGs among five different organs in okra. (**a**) DEGs in different comparisons, including R vs. Fr, S vs. Fr, L vs. Fr, and Fl vs. Fr, are showed by volcanic map. (**b**) Venn diagrams of the DEGs in the four different comparisons. (**c**) GO classification of DEGs in the four different comparisons. From top to down, R vs. Fr comparison, S vs. Fr comparison, L vs. Fr comparison and Fl vs. Fr comparison. R: root; S: stem; L: leaf; Fl: flower; Fr: Fruit.

We then identified differentially expressed unigenes between fruits and any of the other four organs. Based on our analysis, the DEGs in “Fr (fruits) vs. R (roots)”, “Fr vs. S (stems)”, “Fr vs. L (leaves)”, and “Fr vs. Fl (flowers)” were determined to be 22,841, 9883, 45,397, and 53,626, respectively (Figure 4a). In “Fr vs. R”, 7493 upregulated and 15,348 downregulated genes were identified. In “Fr vs. S”, there were 2827 upregulated and 7065 downregulated genes. In fruits compared to leaves, 17,276 upregulated and 28,121 downregulated genes were identified. The comparison between fruits and flowers showed the largest number of differentially expressed genes, with 32,061 genes upregulated and 21,585 downregulated in fruits. A Venn diagram was used to compare the four datasets (Figure 4b). In detail, 1173 DEGs were common to both “Fr vs. R” and “Fr vs. S” comparisons, while 1327 DEGs were identified in both “Fr vs. S” and “Fr vs. L” comparisons. A total of 13,683 DEGs were common to both “Fr vs. L” and “Fr vs. Fl” comparisons, while 497 DEGs were identified in both “Fr vs. Fl” and “Fr vs. R” comparisons. There were 1825 DEGs common to all four comparisons (Figure 4b). GO classifications of DEGs in the four different comparisons were showed in Figure 4c.

To identify the biological functions of the fruit-specifically expressed genes in okra, all DEGs between the fruits and the other organs (Fr vs. R, Fr vs. S, Fr vs. L, and Fr vs. Fl) were subjected to KEGG pathway enrichment analysis. A total of 13,396 DEGs between Fr and R were allocated to 128 pathways, 5,571 DEGs to 121 pathways (Fr vs. S), 26,040 DEGs to 127 pathways (Fr vs. L) and 30,415 DEGs to 128 pathways (Fr vs. Fl) (Tables S2–S5). The ten most highly represented pathways in each comparison are shown in Figure 4c, according to the biological process GO term analysis. GO term analysis indicated that the organ-specific DEGs were involved in various biological processes. Interestingly, three pathways, metabolic pathways, biosynthesis of secondary metabolites and plant–pathogen interaction, were each significantly enriched with respect to DEGs in the R vs. FR, and L vs. FR. Plant–pathogen interaction, biosynthesis of secondary metabolites, and plant hormone signal transduction, were each significantly enriched in DEGs from the S vs. FR comparison, while metabolic pathways, biosynthesis of secondary metabolites, and plant hormone signal transduction, were each significantly enriched in DEGs of the Fl vs. Fr comparison. 

### 3.4. Differentially Expressed Genes Comparison of Total Flavonoids and Polysaccharides in Five Different Organs of Okra

**Figure 5:**
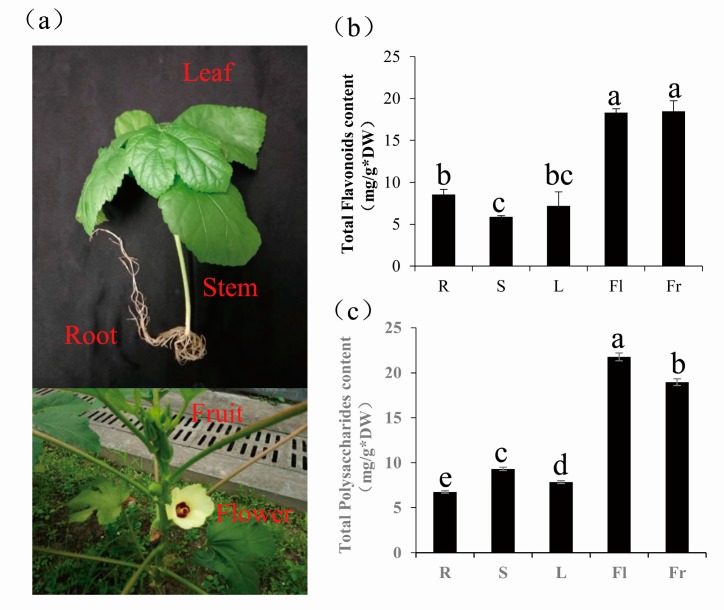
Determination of total flavonoids and polysaccharide contents in five different organs of *Abelmoschus esculentus* (Linn.) Moench. (**a**) Samples from different organs of okra. (**b**) Determination of total flavonoids contents in five organs, including roots, stems, leaves, flowers, and fruits. (**c**) Determination of polysaccharide contents s in five organs, including roots, stems, leaves, flowers, and fruits. Values are ± standard deviations of three independent replicates. Means marked with different letters indicate significant differences at alpha = 0.05 using Duncan’s multiple range tests. DW: dry weight.

The contents of total flavonoids and polysaccharides were determined in all five organs, namely R, S, L, Fl, and Fr, with significant differences in contents being observed between the different organs for both components (Figure 5). Total flavonoids and polysaccharides were mainly concentrated in flowers and fruits (Figure 5b,c). The highest content of total flavonoids was 18.49 mg g^−1^ for fruits, and the highest content of polysaccharides was 21.76 mg g^−1^ for flowers. Using the transcriptome data from the five organs, we explored the molecular mechanism behind of the organ-specific distribution in the active components in okra.

### 3.5. Differentially Expressed Genes Related to Flavonoid Biosynthetic Pathway in Okra

**Figure 6:**
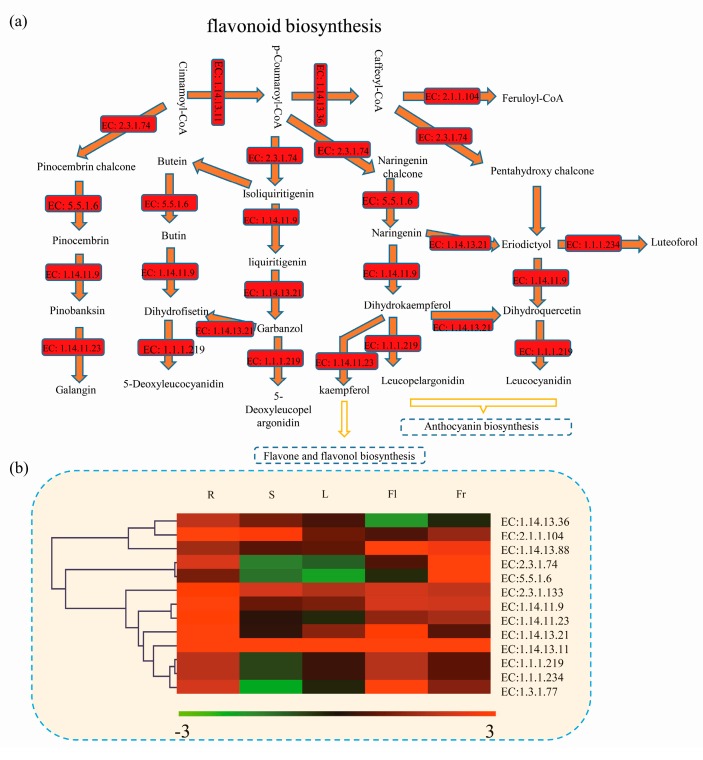
Significance analysis of the flavonoid biosynthesis related unigenes expressions in okra. (**a**) Schematic representation of the enzymes involved in flavonoid biosynthesis pathway. Red indicates significant differentially expressed genes. (**b**) The average expression levels of the unigenes associated with each enzyme were calculated based on FPKM values. EC: 1.14.13.36: coumaroylquinate (coumaroylshikimate) 3′-monooxygenase; EC: 2.1.1.104: caffeoyl-CoA 3-*O*-methyltransferase; EC: 1.14.13.88: flavonoid 3′,5′-hydroxylase; EC: 2.3.1.74: naringenin-chalcone synthase; EC: 5.5.1.6: chalcone isomerase; EC: 2.3.1.133: shikimate *O*-hydroxycinnamoyltransferase; EC: 1.14.11.9: flavanone 3-dioxygenase; EC: 1.14.11.23: flavonol synthase; EC: 1.14.13.21: flavonoid 3′-monooxygenase; EC: 1.14.13.11: trans-cinnamate 4-monooxygenase; EC: 1.1.1.219: dihydrokaempferol 4-reductase; EC: 1.1.1.234: flavanone 4-reductase; EC:1.3.1.77: anthocyanidin reductase. The grids with different colors from green to red show the relative expression levels to log_2_ (FPKM), from −3 to 3. EC: enzyme code.

Most plants contain flavonoids, which play an important role in plant growth, development, flowering, fruit production, and plant defense against pathogens [20]. Total flavonoids were detected in the different parts of the okra (Figure 5b). The biosynthesis pathway of flavonoids has been identified in a number of plant species, which provides us with the opportunity to identify the flavonoid-related enzymes in okra. In total, 931 unigenes encoding 13 flavonoid biosynthesis-related enzymes were identified and quantified in okra (Figure 6). Of these, three enzymes (flavonoid 3',5'-hydroxylase, enzyme code (EC): 1.14.13.88; anthocyanidin reductase, EC: 1.13.1.77; trans-cinnamate 4-monooxygenase, EC: 1.14.13.11) were highly expressed in flowers, while two enzymes (naringenin-chalcone synthase, EC: 2.3.1.74; chalcone isomerase, EC: 5.5.1.6) exhibited high expression levels in fruits and five enzymes (dihydrokaempferol 4-reductase, EC: 1.1.1.219; flavanone 4-reductase, EC: 1.1.1.234; flavanone 3-dioxygenase, EC: 1.14.11.9; flavonoid 30-monooxygenase, EC: 1.14.13.21; naringenin-chalcone synthase, EC: 2.3.1.74) showed lowest expression in stems (Figure 6b). The full names of enzymes by EC IDs were provided in Table S6.

### 3.6. Differentially Expressed Genes Related to Fructose and Mannose Metabolism and Glycosyltransferase Genes in Okra

**Figure 7:**
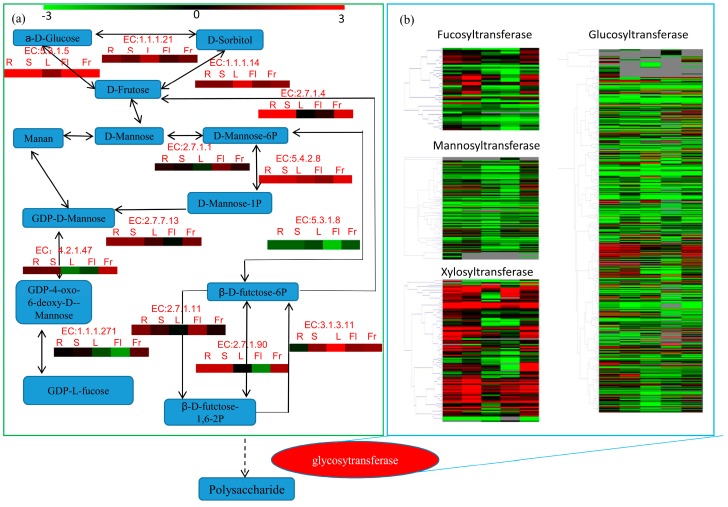
Expression patterns of the putative fructose and mannose metabolism and polysaccharide biosynthesis unigenes in okra. (**A**) The pathway of fructose and mannose metabolism is based on KEGG analysis. The full names of enzymes by EC IDs are provided in Table S7. The average expression level of the enzyme encoding unigenes in various organs is indicated by a heat map. (**B**) Analysis of glycosyltransferase unigenes in four fruit contained comparisons, including R vs. Fr, S vs. Fr, L vs. Fr, and Fl vs. Fr. The grids with different colors from green to red show the relative expression levels to log_2_(FPKM), from −3 to 3. The unigene IDs and the expression level of all glycosyltransferase encoding genes are listed in Table S8.

Our transcriptome data revealed that 691 unigenes encoded 13 key enzymes related to fructose and mannose metabolism. A map of proposed pathways for polysaccharide biosynthesis in okra was constructed (Figure 7a). Unigenes annotated for each enzyme in fructose and mannose metabolism are listed in Table S5. The largest number of unigenes (113) was annotated as aldehyde reductase (EC: 1.1.1.21) encoding genes. The second largest number of unigenes (94) was identified as hexokinase (EC: 2.7.1.1) encoding genes, while 22 unigenes were mannitol-1-phosphate 5-dehydrogenase (EC: 5.4.2.8) encoding gene (Table S7). The expression levels of enzymes were calculated based on average FPKM values of the unigenes (Figure 7a). 

The genes encoding three enzymes, (guanosine diphosphate) GDP-L-fucose synthase (EC: 1.1.1.271), Guanosine diphosphate-mannose 4,6-dehydratase (EC: 4.2.1.47), and phosphomannomutase (EC: 5.4.2.8), were more highly expressed in fruits than in any other okra organs. The biosynthesis of glycan and glycosides requires the participation of glycosyltransferase (GTs; EC: 2.4.x.y) enzymes, which are an important and a very widespread group of enzymes in higher plants [36]. Based on BLASTX [37] comparison of the five transcriptomes, a total of 970 unigenes were annotated to GT genes, consisting of 44 fucosyltransferase genes, 184 mannosyltransferase genes, 104 xylosyltransferase genes, and 638 glucosyltransferase genes. The expression patterns of GT genes were exhibited as a heat map performed by the MeV tool (Figure 7b). The expression levels of four family’s genes were listed in Table S8.

## 4. Discussion

Okra is widely grown as a vegetable, and as a source of herbal medicines in China [38]. Flavonoids and polysaccharides are two of the most important active constituents of okra. In many plants, a large number of genes associated with polysaccharide and flavonoid biosynthesis have been identified [39,40,41], but little is known about the tissue-specific distribution and the molecular basis of biosynthesis of these two active components in okra. The aim of the present study was to identify genes associated with flavonoid and polysaccharide synthesis, and to explain the mechanism of organ-specific distribution of flavonoids and polysaccharides in this plant. 

High-throughput sequencing is suitable for the study of the genome of a non-model plant, which has not been sequenced [42]. Neither the genome size or chromosome count for okra has been precisely reported. Okra (usually 2n = 130) is probably an allotetraploid, derived from a wild species from India (*Abelmoschus tuberculatus* Pal & H.B. Singh, 2n = 58), and a species (possibly *Abelmoschus ficulneus* (L.) Wight & Arn. ex Wight, 2n = 72 [43]). Omics technologies (genomics, transcriptomics, proteomics, and metabolomics) were potential tools to understand the environmental factors influencing okra growth and adaptation [44]. In recent years, some okra transcriptomics data have been published. More than 150,000 unigenes were generated and assembled from the combined leaf and pod transcriptome of okra by Schafleitner’s group [45]. In the present study, 15 transcriptome datasets from five organs of okra, composed of 293,971 unique sequences, were generated and assembled. The number of unigenes generated in our experiments was almost twice that generated by Schafleitner, providing a more comprehensive dataset for the functional study of this species. The fruits (pods or capsules) of okra are rich in dietary fiber, minerals, vitamins, polysaccharides, and flavonoids [20,46]. The detection of genes with fruit-selective expression is a promising strategy for clarifying how polysaccharides and flavonoids accumulate to higher levels in fruits than in other organs of okra. 

In okra, the highest concentration of total flavonoids was in the flowers and fruits (Figure 5b). The expression patterns of the unigenes, annotated as genes related to the flavonoid biosynthesis pathway, were analyzed using the transcriptome data. A large number of DEGs involved in the flavonoid biosynthesis pathway were identified in the present study. Based on the KEGG database, we analyzed the expression pattern of 13 groups of enzymes associated with flavonoid biosynthesis, and found that most of them were downregulated in the stems and leaves, but were upregulated in flowers and fruits (Figure 6b). Many studies have shown that flavonoids play an important role in the growth and germination of the pollen tube [47,48], while anthocyanin accumulation in fruit is an important index of maturity and fruit quality [49]. The flavonoid/phenylpropanoid pathway is responsible for anthocyanin biosynthesis in plants [49,50]. Analysis of the flavonoid synthesis pathway enabled us to understand, at the molecular level, the mechanism by which flavonoids/anthocyanins accumulate in the okra fruit.

RNA sequencing (“RNA-seq”) would facilitate more detailed studies in non-model plants, such as okra. A large number of candidate genes involved in the biosynthesis of polysaccharides were identified in *Polygonatum sibiricum* by transcriptome sequencing [40]. In the present study, three fructose and mannose metabolism-related enzymes, namely GDP-L-fucose synthase, phosphomannomutase, and GDP-mannose 4,6-dehydratase, were shown to be highly expressed in fruits (Figure 7a). GDP-L-fucose synthase (also known as GDP-4-keto-6-deoxy-D-mannose epimerase/reductase, GMER), a key enzyme converting GDP-4-oxo-6-deoxy-D-mannose into GDP-L-fucose, exists widely in animals and plants [51]. Phosphomannomutase (PMM) is a key enzyme in mannose metabolism, which catalyzes the interconversion between mannose-1-phosphate (M1P) and mannose-6-phosphate (M6P) [52]. M1P is the main substrate for the synthesis of GDP-mannose, which is essential for biosynthesis of ascorbic acid (AsA) through the Smirnoff–Wheeler pathway [53,54,55]. Overexpression of the *PMM* gene increased AsA content and tolerance to oxidative stress [53,54], while mild mutations of *PMM* were observed to decrease tolerance to high temperature stress in higher plants [55]. 

The conversion of GDP-D-mannose to GDP-4-keto-6-deoxy-D-mannose is catalyzed by GDP-D-mannose-4, 6-dehydratase (GMD) in a de novo pathway [56]. The *Arabidopsis MUR1* (*GMD2*) gene encodes an isoform of GDP-D-mannose-4,6-dehydratase, catalyzing the dehydration of GDP-D-mannose [15]. It was reported that 95% of N-linked glycans lack L-fucose residues in the defective *Atmur1* mutant, and L-fucose was replaced by a hexose residue in the remaining 5% [57]. Glycosyltransferases (EC 2.4.x.y) exist widely in plants. They are responsible for the catalytic glycosylation from nucleotide-diphospho-sugar (NDP-sugars) to a series of small molecular compounds, forming glycoside compounds, by controlling the formation of glycoside bonds [58,59]. Various NDP-sugars form growing polysaccharide chains by the action of GTs in a key downstream step in polysaccharide biosynthesis [40]. More than 450 members of the GT family were identified in *Arabidopsis*, and over 600 members in rice [36]. In the present study, large numbers of GT-encoding genes were identified as DEGs in the four inter-organ comparisons: Fr vs. R, Fr vs. S, Fr vs. L, and Fr vs. Fl (Figure 7a). In all, 970 unigenes encoding GTs were found in okra (Tables S3 abd S6). By comparison of the FPKM from the 15 RNA-Seq libraries, the most abundant transcript was for glucosyltransferase genes (638), followed by mannosyltransferase genes (184). Our results provide several candidate genes for regulating polysaccharide synthesis and secondary metabolism in okra.

## 5. Conclusions

The content of flavonoids and polysaccharides was determined in different organs of okra, with fruits (highest flavonoid concentration) and flowers (highest polysaccharide concentration) being particularly rich in these compounds. We obtained 15 datasets, comprising 293,971 unigenes, from the okra transcriptome, derived by de novo technology using the Illumina platform. Of these, 232,490 unigenes were annotated by comparison with the Nr, Nt, Swiss-Prot, KEGG, COG, and GO databases. A large number of DEGs were identified in fruits compared with the other four organs. The major biological functions of DEGs were grouped into different GO categories and KEGG pathways. The expression of genes related to the flavonoid biosynthetic pathway, to fructose and mannose metabolism, and to glycosyltransferases, was analyzed in five organs of okra. Our comprehensive transcriptome analysis provides a useful database for further research on this important but little-studied crop.

## Figures and Tables

**Table 1:** Sequencing output statistics of 15 samples.

Samples	Total Raw Reads	Total Clean Reads	Total Clean Nucleotides (nt)	Q20 Percentage	Q30 Percentage	N Percentage	GC Percentage
Root1	48,257,634	46,916,458	7,037,468,700	97.80%	94.49%	0.01%	44.72%
Root2	48,419,454	47,097,672	7,064,650,800	97.92%	94.74%	0.01%	44.86%
Root3	47,422,632	46,112,238	6,916,835,700	97.79%	94.47%	0.00%	44.82%
Stem1	48,647,618	47,316,422	7,097,463,300	97.88%	94.62%	0.01%	44.91%
Stem2	48,693,636	47,323,464	7,098,519,600	97.94%	94.75%	0.01%	44.96%
Stem3	44,275,350	43,132,910	6,469,936,500	97.21%	93.29%	0.00%	44.61%
Leaf1	48,625,474	47,341,802	7,101,270,300	98.00%	94.84%	0.01%	45.61%
Leaf2	47,424,186	46,142,720	6,921,408,000	97.85%	94.52%	0.00%	45.95%
Leaf3	50,618,708	49,272,238	7,390,835,700	97.84%	94.49%	0.00%	45.78%
Flower1	44,078,602	42,777,662	6,416,649,300	97.89%	94.64%	0.00%	45.22%
Flower2	47,743,650	45,883,906	6,882,585,900	97.85%	94.51%	0.00%	45.90%
Flower3	43,314,640	41,960,012	6,294,001,800	97.85%	94.52%	0.00%	45.59%
Fruit1	51,485,706	49,905,802	7,485,870,300	97.45%	93.84%	0.00%	45.26%
Fruit2	49,436,520	48,083,880	7,212,582,000	98.01%	94.90%	0.01%	44.67%
Fruit3	47,886,442	46,568,924	6,985,338,600	97.92%	94.70%	0.00%	44.83%
All	716,330,252	695,836,110	104,375 Gb				

Q20 percentage shows the proportion of nucleotides. Q30 percentage shows the proportion of nucleotides with quality value larger than 30. N: proportion of unknown nucleotides in clean reads.

## Supplementary Materials

The following are available online at http://www.mdpi.com/2073-4425/9/3/130/s1. Table S1: Assembly quality statistics. Table S2: The information of 13,396 significantly DEGs in the “R vs. Fr” comparison were assigned to 128 KEGG pathways. Table S3: The information of 5571 DEGs in “S vs. Fr” comparison were assigned to 121 KEGG pathways. Table S4: The information of 26,040 DEGs in “L vs. Fr” comparison were assigned to 58 KEGG pathways. Table S5: The information of 30,415 DEGs in “Fl vs. Fr” comparison were assigned to 128 KEGG pathways. Table S6: The information of unigenes associated with flavonoid biosynthetic pathway. Table S7: The information of unigenes associated with fructose and mannose metabolism. Table S8: The unigene IDs and the expressional level of all glycosyltransferase encoding genes.
