# Supplementary material for: De Novo Transcriptome Assembly and Characterization of the Synthesis Genes of Bioactive Constituents in Abelmoschus esculentus (L.) Moench
Source: Genes (Basel). 2018 Feb 27;9(3):130. doi: 10.3390/genes9030130 (PMC5867851; doi:10.3390/genes9030130)
Supplement: Supplementary file 1 [file genes-09-00130-s001.zip › Supplemental final/Table S1.docx]

| **Sample** | **Total Number** | **Total Length(nt)** | **Mean Length(nt)** | **N50** | **Total Consensus Sequences** | **Distinct Clusters** | **Distinct Singletons** |
| --- | --- | --- | --- | --- | --- | --- | --- |
| R1 | 142,099 | 93,589,139 | 659 | 1269 | 142,099 | 66,387 | 75,712 |
| R2 | 155,113 | 94,973,183 | 612 | 1157 | 155,113 | 67,131 | 87,982 |
| R3 | 143,370 | 90,440,324 | 631 | 1206 | 143,370 | 64,858 | 78,512 |
| S1 | 141,490 | 96,704,889 | 683 | 1323 | 141,490 | 66,849 | 74,641 |
| S2 | 134,449 | 91,981,530 | 684 | 1326 | 134,449 | 64,414 | 70,035 |
| S3 | 148,107 | 98,171,958 | 663 | 1265 | 148,107 | 69,948 | 78,159 |
| L1 | 120,443 | 81,659,855 | 678 | 1333 | 120,443 | 55,778 | 64,665 |
| L2 | 107,542 | 68,962,884 | 641 | 1253 | 107,542 | 48,564 | 58,978 |
| L3 | 118,344 | 76,998,269 | 651 | 1282 | 118,344 | 53,102 | 65,242 |
| Fl1 | 98,952 | 63,360,220 | 640 | 1254 | 98,952 | 44,085 | 54,867 |
| Fl2 | 105,575 | 68,390,024 | 648 | 1275 | 105,575 | 46,992 | 58,583 |
| Fl3 | 104,282 | 63,108,861 | 605 | 1197 | 104,282 | 41,095 | 63,187 |
| Fr1 | 156,667 | 99,958,213 | 638 | 1198 | 156,667 | 73,653 | 83,014 |
| Fr2 | 140,906 | 91,526,831 | 650 | 1247 | 140,906 | 65,235 | 75,671 |
| Fr3 | 142,708 | 90,357,339 | 633 | 1196 | 142,708 | 66,384 | 76,324 |
| All | 293,971 | 381,392,100 | 1297 | 1885 | 293,971 | 169,409 | 124,562 |

**T**able S1: Assembly quality statistics
