# Supplementary material for: De Novo Transcriptome Assembly and Characterization of the Synthesis Genes of Bioactive Constituents in Abelmoschus esculentus (L.) Moench
Source: Genes (Basel). 2018 Feb 27;9(3):130. doi: 10.3390/genes9030130 (PMC5867851; doi:10.3390/genes9030130)
Supplement: Supplementary file 1 [file genes-09-00130-s001.zip › Supplemental final/Table S2.docx]

**T**able S2: The information of 13,396 significantly DEGs in the “R vs. Fr” comparison were assigned to 128 KEGG pathways

| **Pathway ID** | **Pathway** | **P-value** | **Q-value** |
| --- | --- | --- | --- |
| ko04626 | Plant-pathogen interaction | 1.64E-192 | 2.10E-190 |
| ko01110 | Biosynthesis of secondary metabolites | 2.50E-167 | 1.60E-165 |
| ko00195 | Photosynthesis | 3.08E-132 | 1.32E-130 |
| ko00940 | Phenylpropanoid biosynthesis | 3.55E-124 | 1.14E-122 |
| ko01100 | Metabolic pathways | 6.92E-120 | 1.77E-118 |
| ko00941 | Flavonoid biosynthesis | 4.38E-107 | 9.35E-106 |
| ko00945 | Stilbenoid, diarylheptanoid and gingerol biosynthesis | 3.41E-92 | 6.24E-91 |
| ko00196 | Photosynthesis - antenna proteins | 2.49E-61 | 3.98E-60 |
| ko00944 | Flavone and flavonol biosynthesis | 3.35E-59 | 4.76E-58 |
| ko04075 | Plant hormone signal transduction | 4.17E-57 | 5.34E-56 |
| ko00360 | Phenylalanine metabolism | 6.88E-53 | 8.01E-52 |
| ko02010 | ABC transporters | 1.21E-48 | 1.29E-47 |
| ko00903 | Limonene and pinene degradation | 1.89E-36 | 1.86E-35 |
| ko00430 | Taurine and hypotaurine metabolism | 3.32E-36 | 3.03E-35 |
| ko00073 | Cutin, suberine and wax biosynthesis | 2.18E-35 | 1.86E-34 |
| ko00904 | Diterpenoid biosynthesis | 2.34E-32 | 1.87E-31 |
| ko00908 | Zeatin biosynthesis | 2.60E-30 | 1.95E-29 |
| ko00710 | Carbon fixation in photosynthetic organisms | 5.18E-30 | 3.69E-29 |
| ko00592 | alpha-Linolenic acid metabolism | 3.33E-28 | 2.24E-27 |
| ko00906 | Carotenoid biosynthesis | 4.74E-28 | 3.03E-27 |
| ko00905 | Brassinosteroid biosynthesis | 3.82E-26 | 2.33E-25 |
| ko00130 | Ubiquinone and other terpenoid-quinone biosynthesis | 9.10E-23 | 5.30E-22 |
| ko00250 | Alanine, aspartate and glutamate metabolism | 1.31E-19 | 7.30E-19 |
| ko00650 | Butanoate metabolism | 1.79E-19 | 9.52E-19 |
| ko00053 | Ascorbate and aldarate metabolism | 2.54E-16 | 1.30E-15 |
| ko00943 | Isoflavonoid biosynthesis | 1.93E-15 | 9.52E-15 |
| ko00750 | Vitamin B6 metabolism | 2.09E-13 | 9.89E-13 |
| ko00071 | Fatty acid metabolism | 4.04E-13 | 1.85E-12 |
| ko00564 | Glycerophospholipid metabolism | 3.33E-12 | 1.47E-11 |
| ko00402 | Benzoxazinoid biosynthesis | 1.18E-11 | 5.02E-11 |
| ko00565 | Ether lipid metabolism | 1.82E-11 | 7.50E-11 |
| ko00410 | beta-Alanine metabolism | 2.03E-11 | 8.12E-11 |
| ko00511 | Other glycan degradation | 4.04E-11 | 1.57E-10 |
| ko00480 | Glutathione metabolism | 4.15E-10 | 1.56E-09 |
| ko00380 | Tryptophan metabolism | 7.68E-10 | 2.81E-09 |
| ko00966 | Glucosinolate biosynthesis | 1.23E-09 | 4.38E-09 |
| ko00902 | Monoterpenoid biosynthesis | 5.23E-08 | 1.81E-07 |
| ko00909 | Sesquiterpenoid and triterpenoid biosynthesis | 7.08E-08 | 2.38E-07 |
| ko00620 | Pyruvate metabolism | 4.28E-07 | 1.40E-06 |
| ko00030 | Pentose phosphate pathway | 7.19E-07 | 2.30E-06 |
| ko00910 | Nitrogen metabolism | 1.15E-06 | 3.60E-06 |
| ko01040 | Biosynthesis of unsaturated fatty acids | 1.75E-05 | 5.34E-05 |
| ko00900 | Terpenoid backbone biosynthesis | 0.00012029 | 3.58E-04 |
| ko00860 | Porphyrin and chlorophyll metabolism | 0.000265703 | 7.73E-04 |
| ko04650 | Natural killer cell mediated cytotoxicity | 0.000442368 | 1.26E-03 |
| ko00500 | Starch and sucrose metabolism | 0.001086375 | 3.02E-03 |
| ko00010 | Glycolysis / Gluconeogenesis | 0.001874091 | 5.10E-03 |
| ko00062 | Fatty acid elongation | 0.002141963 | 5.71E-03 |
| ko00040 | Pentose and glucuronate interconversions | 0.003573279 | 9.33E-03 |
| ko00950 | Isoquinoline alkaloid biosynthesis | 0.003800082 | 9.73E-03 |
| ko04130 | SNARE interactions in vesicular transport | 0.004447916 | 1.12E-02 |
| ko00052 | Galactose metabolism | 0.01209056 | 2.98E-02 |
| ko00901 | Indole alkaloid biosynthesis | 0.01787236 | 4.32E-02 |
| ko00942 | Anthocyanin biosynthesis | 0.02018622 | 4.78E-02 |
| ko00350 | Tyrosine metabolism | 0.03110797 | 7.24E-02 |
| ko00051 | Fructose and mannose metabolism | 0.03209014 | 7.33E-02 |
| ko00630 | Glyoxylate and dicarboxylate metabolism | 0.06697356 | 1.50E-01 |
| ko00591 | Linoleic acid metabolism | 0.08913553 | 1.97E-01 |
| ko00460 | Cyanoamino acid metabolism | 0.09218096 | 2.00E-01 |
| ko00603 | Glycosphingolipid biosynthesis - globo series | 0.1400884 | 2.99E-01 |
| ko00965 | Betalain biosynthesis | 0.1428772 | 3.00E-01 |
| ko00563 | Glycosylphosphatidylinositol(GPI)-anchor biosynthesis | 0.1813458 | 3.74E-01 |
| ko00280 | Valine, leucine and isoleucine degradation | 0.18415 | 3.74E-01 |
| ko00561 | Glycerolipid metabolism | 0.2247231 | 4.49E-01 |
| ko04146 | Peroxisome | 0.2718665 | 5.35E-01 |
| ko00960 | Tropane, piperidine and pyridine alkaloid biosynthesis | 0.2908334 | 5.64E-01 |
| ko04144 | Endocytosis | 0.3357361 | 6.41E-01 |
| ko00270 | Cysteine and methionine metabolism | 0.3703103 | 6.97E-01 |
| ko00072 | Synthesis and degradation of ketone bodies | 0.4010706 | 7.44E-01 |
| ko00660 | C5-Branched dibasic acid metabolism | 0.555855 | 1.00E+00 |
| ko04070 | Phosphatidylinositol signaling system | 0.6331728 | 1.00E+00 |
| ko00640 | Propanoate metabolism | 0.6407375 | 1.00E+00 |
| ko00780 | Biotin metabolism | 0.7314649 | 1.00E+00 |
| ko00920 | Sulfur metabolism | 0.7595593 | 1.00E+00 |
| ko00604 | Glycosphingolipid biosynthesis - ganglio series | 0.7608784 | 1.00E+00 |
| ko00740 | Riboflavin metabolism | 0.8101089 | 1.00E+00 |
| ko00260 | Glycine, serine and threonine metabolism | 0.8439769 | 1.00E+00 |
| ko00600 | Sphingolipid metabolism | 0.8514398 | 1.00E+00 |
| ko00232 | Caffeine metabolism | 0.8958481 | 1.00E+00 |
| ko03020 | RNA polymerase | 0.9103702 | 1.00E+00 |
| ko00590 | Arachidonic acid metabolism | 0.94027 | 1.00E+00 |
| ko00785 | Lipoic acid metabolism | 0.949074 | 1.00E+00 |
| ko00514 | Other types of O-glycan biosynthesis | 0.9570185 | 1.00E+00 |
| ko00340 | Histidine metabolism | 0.9580527 | 1.00E+00 |
| ko00450 | Selenocompound metabolism | 0.9678816 | 1.00E+00 |
| ko00770 | Pantothenate and CoA biosynthesis | 0.9783636 | 1.00E+00 |
| ko00290 | Valine, leucine and isoleucine biosynthesis | 0.988281 | 1.00E+00 |
| ko04712 | Circadian rhythm - plant | 0.9925384 | 1.00E+00 |
| ko00562 | Inositol phosphate metabolism | 0.995145 | 1.00E+00 |
| ko00061 | Fatty acid biosynthesis | 0.9954273 | 1.00E+00 |
| ko00730 | Thiamine metabolism | 0.9956972 | 1.00E+00 |
| ko00790 | Folate biosynthesis | 0.9962289 | 1.00E+00 |
| ko00310 | Lysine degradation | 0.9970452 | 1.00E+00 |
| ko00300 | Lysine biosynthesis | 0.9982239 | 1.00E+00 |
| ko00531 | Glycosaminoglycan degradation | 0.998594 | 1.00E+00 |
| ko00190 | Oxidative phosphorylation | 0.9991594 | 1.00E+00 |
| ko03410 | Base excision repair | 0.9993887 | 1.00E+00 |
| ko00520 | Amino sugar and nucleotide sugar metabolism | 0.9996232 | 1.00E+00 |
| ko00670 | One carbon pool by folate | 0.9996234 | 1.00E+00 |
| ko00400 | Phenylalanine, tyrosine and tryptophan biosynthesis | 0.9996324 | 1.00E+00 |
| ko00020 | Citrate cycle (TCA cycle) | 0.9997973 | 1.00E+00 |
| ko04140 | Regulation of autophagy | 0.9998549 | 1.00E+00 |
| ko00760 | Nicotinate and nicotinamide metabolism | 0.9998791 | 1.00E+00 |
| ko03450 | Non-homologous end-joining | 0.9998791 | 1.00E+00 |
| ko00330 | Arginine and proline metabolism | 0.9999055 | 1.00E+00 |
| ko00100 | Steroid biosynthesis | 0.9999508 | 1.00E+00 |
| ko04122 | Sulfur relay system | 0.9999927 | 1.00E+00 |
| ko03030 | DNA replication | 0.9999996 | 1.00E+00 |
| ko04710 | Circadian rhythm - mammal | 1 | 1.00E+00 |
| ko03060 | Protein export | 1 | 1.00E+00 |
| ko03050 | Proteasome | 1 | 1.00E+00 |
| ko00230 | Purine metabolism | 1 | 1.00E+00 |
| ko03430 | Mismatch repair | 1 | 1.00E+00 |
| ko03010 | Ribosome | 1 | 1.00E+00 |
| ko04120 | Ubiquitin mediated proteolysis | 1 | 1.00E+00 |
| ko04141 | Protein processing in endoplasmic reticulum | 1 | 1.00E+00 |
| ko03440 | Homologous recombination | 1 | 1.00E+00 |
| ko03420 | Nucleotide excision repair | 1 | 1.00E+00 |
| ko00510 | N-Glycan biosynthesis | 1 | 1.00E+00 |
| ko03022 | Basal transcription factors | 1 | 1.00E+00 |
| ko03015 | mRNA surveillance pathway | 1 | 1.00E+00 |
| ko03040 | Spliceosome | 1 | 1.00E+00 |
| ko03013 | RNA transport | 1 | 1.00E+00 |
| ko00970 | Aminoacyl-tRNA biosynthesis | 1 | 1.00E+00 |
| ko03008 | Ribosome biogenesis in eukaryotes | 1 | 1.00E+00 |
| ko04145 | Phagosome | 1 | 1.00E+00 |
| ko03018 | RNA degradation | 1 | 1.00E+00 |
| ko00240 | Pyrimidine metabolism | 1 | 1.00E+00 |
