# Supplementary material for: De Novo Transcriptome Assembly and Characterization of the Synthesis Genes of Bioactive Constituents in Abelmoschus esculentus (L.) Moench
Source: Genes (Basel). 2018 Feb 27;9(3):130. doi: 10.3390/genes9030130 (PMC5867851; doi:10.3390/genes9030130)
Supplement: Supplementary file 1 [file genes-09-00130-s001.zip › Supplemental final/Table S3.docx]

**T**able S3: The information of 5,571 DEGs in “S vs Fr” comparison were assigned to 121 KEGG pathways

| **Pathway ID** | **Pathway** | **Pvalue** | **Qvalue** |
| --- | --- | --- | --- |
| ko04626 | Plant-pathogen interaction | 2.04E-159 | 2.47E-157 |
| ko00941 | Flavonoid biosynthesis | 3.45E-58 | 2.09E-56 |
| ko04075 | Plant hormone signal transduction | 1.05E-39 | 4.23E-38 |
| ko01110 | Biosynthesis of secondary metabolites | 4.66E-35 | 1.41E-33 |
| ko00940 | Phenylpropanoid biosynthesis | 1.17E-30 | 2.83E-29 |
| ko00945 | Stilbenoid, diarylheptanoid and gingerol biosynthesis | 4.88E-29 | 9.84E-28 |
| ko00944 | Flavone and flavonol biosynthesis | 2.40E-25 | 3.63E-24 |
| ko04650 | Natural killer cell mediated cytotoxicity | 2.40E-25 | 3.63E-24 |
| ko00430 | Taurine and hypotaurine metabolism | 1.42E-18 | 1.91E-17 |
| ko00062 | Fatty acid elongation | 3.94E-18 | 4.76E-17 |
| ko00073 | Cutin, suberine and wax biosynthesis | 1.83E-17 | 2.02E-16 |
| ko00906 | Carotenoid biosynthesis | 1.80E-11 | 1.81E-10 |
| ko00053 | Ascorbate and aldarate metabolism | 3.90E-11 | 3.63E-10 |
| ko00903 | Limonene and pinene degradation | 2.63E-09 | 2.28E-08 |
| ko00750 | Vitamin B6 metabolism | 1.82E-07 | 1.46E-06 |
| ko00511 | Other glycan degradation | 7.42E-06 | 5.61E-05 |
| ko01100 | Metabolic pathways | 1.30E-05 | 9.25E-05 |
| ko00901 | Indole alkaloid biosynthesis | 2.37E-05 | 1.59E-04 |
| ko00650 | Butanoate metabolism | 5.03E-05 | 3.21E-04 |
| ko00500 | Starch and sucrose metabolism | 0.000167895 | 1.02E-03 |
| ko04140 | Regulation of autophagy | 0.000208017 | 1.15E-03 |
| ko00520 | Amino sugar and nucleotide sugar metabolism | 0.000209061 | 1.15E-03 |
| ko02010 | ABC transporters | 0.000231377 | 1.22E-03 |
| ko00270 | Cysteine and methionine metabolism | 0.000254893 | 1.29E-03 |
| ko00410 | beta-Alanine metabolism | 0.000286462 | 1.39E-03 |
| ko00402 | Benzoxazinoid biosynthesis | 0.00045401 | 2.11E-03 |
| ko00360 | Phenylalanine metabolism | 0.000911148 | 4.08E-03 |
| ko00040 | Pentose and glucuronate interconversions | 0.001260943 | 5.45E-03 |
| ko00966 | Glucosinolate biosynthesis | 0.002618752 | 1.09E-02 |
| ko00908 | Zeatin biosynthesis | 0.002744689 | 1.11E-02 |
| ko00250 | Alanine, aspartate and glutamate metabolism | 0.003689774 | 1.44E-02 |
| ko00904 | Diterpenoid biosynthesis | 0.007536667 | 2.85E-02 |
| ko00561 | Glycerolipid metabolism | 0.008743177 | 3.21E-02 |
| ko00592 | alpha-Linolenic acid metabolism | 0.01599481 | 5.69E-02 |
| ko00905 | Brassinosteroid biosynthesis | 0.02419007 | 8.36E-02 |
| ko00902 | Monoterpenoid biosynthesis | 0.03731459 | 1.25E-01 |
| ko04130 | SNARE interactions in vesicular transport | 0.03825497 | 1.25E-01 |
| ko00460 | Cyanoamino acid metabolism | 0.04398605 | 1.40E-01 |
| ko00910 | Nitrogen metabolism | 0.05103981 | 1.58E-01 |
| ko00942 | Anthocyanin biosynthesis | 0.1502554 | 4.55E-01 |
| ko00591 | Linoleic acid metabolism | 0.1642498 | 4.85E-01 |
| ko00780 | Biotin metabolism | 0.1997599 | 5.75E-01 |
| ko00943 | Isoflavonoid biosynthesis | 0.2323975 | 6.54E-01 |
| ko03020 | RNA polymerase | 0.2376985 | 6.54E-01 |
| ko00600 | Sphingolipid metabolism | 0.2809224 | 7.55E-01 |
| ko00563 | Glycosylphosphatidylinositol(GPI)-anchor biosynthesis | 0.3140179 | 8.26E-01 |
| ko00909 | Sesquiterpenoid and triterpenoid biosynthesis | 0.3269786 | 8.42E-01 |
| ko00920 | Sulfur metabolism | 0.4342978 | 1.00E+00 |
| ko00450 | Selenocompound metabolism | 0.4426962 | 1.00E+00 |
| ko04070 | Phosphatidylinositol signaling system | 0.4437059 | 1.00E+00 |
| ko00660 | C5-Branched dibasic acid metabolism | 0.453209 | 1.00E+00 |
| ko00565 | Ether lipid metabolism | 0.4691855 | 1.00E+00 |
| ko00380 | Tryptophan metabolism | 0.4898263 | 1.00E+00 |
| ko00640 | Propanoate metabolism | 0.5600153 | 1.00E+00 |
| ko00232 | Caffeine metabolism | 0.5985157 | 1.00E+00 |
| ko00071 | Fatty acid metabolism | 0.7035511 | 1.00E+00 |
| ko00052 | Galactose metabolism | 0.7098725 | 1.00E+00 |
| ko00300 | Lysine biosynthesis | 0.7119884 | 1.00E+00 |
| ko00061 | Fatty acid biosynthesis | 0.7487127 | 1.00E+00 |
| ko00564 | Glycerophospholipid metabolism | 0.7603637 | 1.00E+00 |
| ko00950 | Isoquinoline alkaloid biosynthesis | 0.7906435 | 1.00E+00 |
| ko00604 | Glycosphingolipid biosynthesis - ganglio series | 0.8258579 | 1.00E+00 |
| ko01040 | Biosynthesis of unsaturated fatty acids | 0.8612093 | 1.00E+00 |
| ko04712 | Circadian rhythm - plant | 0.8971941 | 1.00E+00 |
| ko00350 | Tyrosine metabolism | 0.9155051 | 1.00E+00 |
| ko00740 | Riboflavin metabolism | 0.9180091 | 1.00E+00 |
| ko00860 | Porphyrin and chlorophyll metabolism | 0.918688 | 1.00E+00 |
| ko00514 | Other types of O-glycan biosynthesis | 0.9329103 | 1.00E+00 |
| ko00030 | Pentose phosphate pathway | 0.935005 | 1.00E+00 |
| ko03450 | Non-homologous end-joining | 0.9518867 | 1.00E+00 |
| ko00130 | Ubiquinone and other terpenoid-quinone biosynthesis | 0.9625186 | 1.00E+00 |
| ko00196 | Photosynthesis - antenna proteins | 0.9635579 | 1.00E+00 |
| ko00603 | Glycosphingolipid biosynthesis - globo series | 0.9729411 | 1.00E+00 |
| ko00562 | Inositol phosphate metabolism | 0.9743079 | 1.00E+00 |
| ko00340 | Histidine metabolism | 0.9820167 | 1.00E+00 |
| ko00510 | N-Glycan biosynthesis | 0.984628 | 1.00E+00 |
| ko04145 | Phagosome | 0.9867399 | 1.00E+00 |
| ko00760 | Nicotinate and nicotinamide metabolism | 0.9873793 | 1.00E+00 |
| ko00590 | Arachidonic acid metabolism | 0.9888661 | 1.00E+00 |
| ko00531 | Glycosaminoglycan degradation | 0.9901834 | 1.00E+00 |
| ko00900 | Terpenoid backbone biosynthesis | 0.9915117 | 1.00E+00 |
| ko00290 | Valine, leucine and isoleucine biosynthesis | 0.9946554 | 1.00E+00 |
| ko00480 | Glutathione metabolism | 0.996228 | 1.00E+00 |
| ko03440 | Homologous recombination | 0.9970534 | 1.00E+00 |
| ko04146 | Peroxisome | 0.9988639 | 1.00E+00 |
| ko00240 | Pyrimidine metabolism | 0.9989755 | 1.00E+00 |
| ko00620 | Pyruvate metabolism | 0.9992358 | 1.00E+00 |
| ko00230 | Purine metabolism | 0.9997257 | 1.00E+00 |
| ko00770 | Pantothenate and CoA biosynthesis | 0.9997764 | 1.00E+00 |
| ko00260 | Glycine, serine and threonine metabolism | 0.9998036 | 1.00E+00 |
| ko00960 | Tropane, piperidine and pyridine alkaloid biosynthesis | 0.9998436 | 1.00E+00 |
| ko03430 | Mismatch repair | 0.9998775 | 1.00E+00 |
| ko00710 | Carbon fixation in photosynthetic organisms | 0.9998925 | 1.00E+00 |
| ko00280 | Valine, leucine and isoleucine degradation | 0.9998938 | 1.00E+00 |
| ko00100 | Steroid biosynthesis | 0.9999431 | 1.00E+00 |
| ko03410 | Base excision repair | 0.9999731 | 1.00E+00 |
| ko04144 | Endocytosis | 0.999984 | 1.00E+00 |
| ko03030 | DNA replication | 0.9999845 | 1.00E+00 |
| ko00195 | Photosynthesis | 0.9999946 | 1.00E+00 |
| ko00310 | Lysine degradation | 0.9999974 | 1.00E+00 |
| ko03420 | Nucleotide excision repair | 0.9999987 | 1.00E+00 |
| ko04710 | Circadian rhythm - mammal | 0.9999992 | 1.00E+00 |
| ko00020 | Citrate cycle (TCA cycle) | 0.9999995 | 1.00E+00 |
| ko00630 | Glyoxylate and dicarboxylate metabolism | 0.9999997 | 1.00E+00 |
| ko00400 | Phenylalanine, tyrosine and tryptophan biosynthesis | 0.9999998 | 1.00E+00 |
| ko03060 | Protein export | 0.9999998 | 1.00E+00 |
| ko03022 | Basal transcription factors | 0.9999999 | 1.00E+00 |
| ko00051 | Fructose and mannose metabolism | 0.9999999 | 1.00E+00 |
| ko00010 | Glycolysis / Gluconeogenesis | 1 | 1.00E+00 |
| ko03015 | mRNA surveillance pathway | 1 | 1.00E+00 |
| ko03050 | Proteasome | 1 | 1.00E+00 |
| ko00190 | Oxidative phosphorylation | 1 | 1.00E+00 |
| ko03018 | RNA degradation | 1 | 1.00E+00 |
| ko04141 | Protein processing in endoplasmic reticulum | 1 | 1.00E+00 |
| ko03040 | Spliceosome | 1 | 1.00E+00 |
| ko00970 | Aminoacyl-tRNA biosynthesis | 1 | 1.00E+00 |
| ko00330 | Arginine and proline metabolism | 1 | 1.00E+00 |
| ko03008 | Ribosome biogenesis in eukaryotes | 1 | 1.00E+00 |
| ko03010 | Ribosome | 1 | 1.00E+00 |
| ko03013 | RNA transport | 1 | 1.00E+00 |
| ko04120 | Ubiquitin mediated proteolysis | 1 | 1.00E+00 |
