# Supplementary material for: De Novo Transcriptome Assembly and Characterization of the Synthesis Genes of Bioactive Constituents in Abelmoschus esculentus (L.) Moench
Source: Genes (Basel). 2018 Feb 27;9(3):130. doi: 10.3390/genes9030130 (PMC5867851; doi:10.3390/genes9030130)
Supplement: Supplementary file 1 [file genes-09-00130-s001.zip › Supplemental final/Table S4.docx]

**T**able S4: The information of 26,040 DEGs in “L vs Fr” comparison were assigned to 58 KEGG pathways

| **Pathway ID** | **Pathway** | **Pvalue** | **Qvalue** |
| --- | --- | --- | --- |
| ko04626 | Plant-pathogen interaction | 2.05E-113 | 2.61E-111 |
| ko00195 | Photosynthesis | 1.79E-110 | 1.14E-108 |
| ko01110 | Biosynthesis of secondary metabolites | 2.23E-76 | 9.46E-75 |
| ko04075 | Plant hormone signal transduction | 5.42E-65 | 1.72E-63 |
| ko00196 | Photosynthesis - antenna proteins | 5.41E-59 | 1.37E-57 |
| ko01100 | Metabolic pathways | 7.43E-49 | 1.57E-47 |
| ko00906 | Carotenoid biosynthesis | 6.31E-45 | 1.15E-43 |
| ko00941 | Flavonoid biosynthesis | 4.08E-33 | 6.47E-32 |
| ko00944 | Flavone and flavonol biosynthesis | 3.75E-32 | 5.29E-31 |
| ko04712 | Circadian rhythm - plant | 1.01E-28 | 1.28E-27 |
| ko00710 | Carbon fixation in photosynthetic organisms | 1.90E-26 | 2.19E-25 |
| ko00945 | Stilbenoid, diarylheptanoid and gingerol biosynthesis | 1.13E-25 | 1.19E-24 |
| ko00903 | Limonene and pinene degradation | 1.06E-24 | 1.03E-23 |
| ko00430 | Taurine and hypotaurine metabolism | 6.39E-23 | 5.80E-22 |
| ko00051 | Fructose and mannose metabolism | 1.49E-19 | 1.26E-18 |
| ko00010 | Glycolysis / Gluconeogenesis | 9.52E-16 | 7.56E-15 |
| ko00030 | Pentose phosphate pathway | 1.89E-14 | 1.41E-13 |
| ko00860 | Porphyrin and chlorophyll metabolism | 7.68E-14 | 5.42E-13 |
| ko00905 | Brassinosteroid biosynthesis | 2.31E-13 | 1.55E-12 |
| ko00053 | Ascorbate and aldarate metabolism | 8.73E-13 | 5.54E-12 |
| ko00500 | Starch and sucrose metabolism | 5.78E-11 | 3.45E-10 |
| ko00561 | Glycerolipid metabolism | 5.98E-11 | 3.45E-10 |
| ko03010 | Ribosome | 7.09E-11 | 3.91E-10 |
| ko00909 | Sesquiterpenoid and triterpenoid biosynthesis | 1.59E-10 | 8.42E-10 |
| ko00591 | Linoleic acid metabolism | 5.41E-09 | 2.75E-08 |
| ko00650 | Butanoate metabolism | 6.14E-09 | 3.00E-08 |
| ko00630 | Glyoxylate and dicarboxylate metabolism | 2.50E-08 | 1.18E-07 |
| ko00750 | Vitamin B6 metabolism | 5.57E-08 | 2.52E-07 |
| ko00740 | Riboflavin metabolism | 1.28E-07 | 5.59E-07 |
| ko02010 | ABC transporters | 1.79E-07 | 7.57E-07 |
| ko00910 | Nitrogen metabolism | 3.50E-06 | 1.44E-05 |
| ko00511 | Other glycan degradation | 3.94E-06 | 1.56E-05 |
| ko00904 | Diterpenoid biosynthesis | 5.43E-06 | 2.09E-05 |
| ko00604 | Glycosphingolipid biosynthesis - ganglio series | 7.86E-06 | 2.94E-05 |
| ko00966 | Glucosinolate biosynthesis | 9.50E-06 | 3.43E-05 |
| ko00940 | Phenylpropanoid biosynthesis | 9.71E-06 | 3.43E-05 |
| ko00250 | Alanine, aspartate and glutamate metabolism | 1.14E-05 | 3.91E-05 |
| ko00062 | Fatty acid elongation | 2.12E-05 | 7.09E-05 |
| ko00380 | Tryptophan metabolism | 6.49E-05 | 2.11E-04 |
| ko00592 | alpha-Linolenic acid metabolism | 0.0001311 | 4.16E-04 |
| ko00130 | Ubiquinone and other terpenoid-quinone biosynthesis | 0.0001523 | 4.72E-04 |
| ko00908 | Zeatin biosynthesis | 0.0002279 | 6.89E-04 |
| ko00410 | beta-Alanine metabolism | 0.0004864 | 1.41E-03 |
| ko00040 | Pentose and glucuronate interconversions | 0.0004896 | 1.41E-03 |
| ko00260 | Glycine, serine and threonine metabolism | 0.0017699 | 4.99E-03 |
| ko00590 | Arachidonic acid metabolism | 0.0022402 | 6.18E-03 |
| ko00900 | Terpenoid backbone biosynthesis | 0.0028609 | 7.73E-03 |
| ko00402 | Benzoxazinoid biosynthesis | 0.0030838 | 8.16E-03 |
| ko00300 | Lysine biosynthesis | 0.0039517 | 1.02E-02 |
| ko00902 | Monoterpenoid biosynthesis | 0.0078638 | 2.00E-02 |
| ko04070 | Phosphatidylinositol signaling system | 0.0084253 | 2.10E-02 |
| ko00785 | Lipoic acid metabolism | 0.0089013 | 2.17E-02 |
| ko00052 | Galactose metabolism | 0.0110258 | 2.64E-02 |
| ko00920 | Sulfur metabolism | 0.013366 | 3.14E-02 |
| ko00460 | Cyanoamino acid metabolism | 0.0382899 | 8.84E-02 |
| ko00480 | Glutathione metabolism | 0.0414518 | 9.40E-02 |
| ko00520 | Amino sugar and nucleotide sugar metabolism | 0.0427587 | 9.53E-02 |
| ko00531 | Glycosaminoglycan degradation | 0.0466549 | 1.02E-01 |
| ko00901 | Indole alkaloid biosynthesis | 0.0651716 | 1.40E-01 |
| ko03060 | Protein export | 0.0704079 | 1.49E-01 |
| ko00942 | Anthocyanin biosynthesis | 0.0862672 | 1.80E-01 |
| ko00073 | Cutin, suberine and wax biosynthesis | 0.1029012 | 2.11E-01 |
| ko00450 | Selenocompound metabolism | 0.1090594 | 2.20E-01 |
| ko00072 | Synthesis and degradation of ketone bodies | 0.1428307 | 2.83E-01 |
| ko04140 | Regulation of autophagy | 0.1543784 | 3.02E-01 |
| ko00660 | C5-Branched dibasic acid metabolism | 0.1694411 | 3.26E-01 |
| ko03410 | Base excision repair | 0.1855277 | 3.52E-01 |
| ko00340 | Histidine metabolism | 0.1974508 | 3.69E-01 |
| ko00943 | Isoflavonoid biosynthesis | 0.2475555 | 4.56E-01 |
| ko00562 | Inositol phosphate metabolism | 0.2695648 | 4.89E-01 |
| ko00760 | Nicotinate and nicotinamide metabolism | 0.3157077 | 5.65E-01 |
| ko00600 | Sphingolipid metabolism | 0.3932399 | 6.94E-01 |
| ko03030 | DNA replication | 0.4902568 | 8.53E-01 |
| ko03020 | RNA polymerase | 0.5799615 | 9.95E-01 |
| ko04650 | Natural killer cell mediated cytotoxicity | 0.6076202 | 1.00E+00 |
| ko00730 | Thiamine metabolism | 0.6109639 | 1.00E+00 |
| ko00780 | Biotin metabolism | 0.7540607 | 1.00E+00 |
| ko00280 | Valine, leucine and isoleucine degradation | 0.7768447 | 1.00E+00 |
| ko01040 | Biosynthesis of unsaturated fatty acids | 0.7938263 | 1.00E+00 |
| ko00270 | Cysteine and methionine metabolism | 0.7964524 | 1.00E+00 |
| ko00071 | Fatty acid metabolism | 0.8057371 | 1.00E+00 |
| ko00564 | Glycerophospholipid metabolism | 0.8170713 | 1.00E+00 |
| ko00620 | Pyruvate metabolism | 0.8180982 | 1.00E+00 |
| ko04130 | SNARE interactions in vesicular transport | 0.8229934 | 1.00E+00 |
| ko00565 | Ether lipid metabolism | 0.8260425 | 1.00E+00 |
| ko00603 | Glycosphingolipid biosynthesis - globo series | 0.8660131 | 1.00E+00 |
| ko00100 | Steroid biosynthesis | 0.8833762 | 1.00E+00 |
| ko00670 | One carbon pool by folate | 0.8880203 | 1.00E+00 |
| ko00360 | Phenylalanine metabolism | 0.9380578 | 1.00E+00 |
| ko03430 | Mismatch repair | 0.9492337 | 1.00E+00 |
| ko00640 | Propanoate metabolism | 0.9548707 | 1.00E+00 |
| ko00790 | Folate biosynthesis | 0.9851377 | 1.00E+00 |
| ko00061 | Fatty acid biosynthesis | 0.9862745 | 1.00E+00 |
| ko00965 | Betalain biosynthesis | 0.9880041 | 1.00E+00 |
| ko03450 | Non-homologous end-joining | 0.9945252 | 1.00E+00 |
| ko00770 | Pantothenate and CoA biosynthesis | 0.9947113 | 1.00E+00 |
| ko04145 | Phagosome | 0.9952093 | 1.00E+00 |
| ko00290 | Valine, leucine and isoleucine biosynthesis | 0.9955191 | 1.00E+00 |
| ko00563 | Glycosylphosphatidylinositol(GPI)-anchor biosynthesis | 0.996331 | 1.00E+00 |
| ko03440 | Homologous recombination | 0.9966619 | 1.00E+00 |
| ko04120 | Ubiquitin mediated proteolysis | 0.9976765 | 1.00E+00 |
| ko00514 | Other types of O-glycan biosynthesis | 0.9980231 | 1.00E+00 |
| ko00330 | Arginine and proline metabolism | 0.9991506 | 1.00E+00 |
| ko00960 | Tropane, piperidine and pyridine alkaloid biosynthesis | 0.9994817 | 1.00E+00 |
| ko04122 | Sulfur relay system | 0.9997678 | 1.00E+00 |
| ko00350 | Tyrosine metabolism | 0.9997835 | 1.00E+00 |
| ko00230 | Purine metabolism | 0.999828 | 1.00E+00 |
| ko04146 | Peroxisome | 0.9999411 | 1.00E+00 |
| ko00950 | Isoquinoline alkaloid biosynthesis | 0.9999787 | 1.00E+00 |
| ko00310 | Lysine degradation | 0.9999921 | 1.00E+00 |
| ko00970 | Aminoacyl-tRNA biosynthesis | 0.9999945 | 1.00E+00 |
| ko03420 | Nucleotide excision repair | 0.9999985 | 1.00E+00 |
| ko00400 | Phenylalanine, tyrosine and tryptophan biosynthesis | 0.9999998 | 1.00E+00 |
| ko03015 | mRNA surveillance pathway | 1 | 1.00E+00 |
| ko04710 | Circadian rhythm - mammal | 1 | 1.00E+00 |
| ko03050 | Proteasome | 1 | 1.00E+00 |
| ko00190 | Oxidative phosphorylation | 1 | 1.00E+00 |
| ko00020 | Citrate cycle (TCA cycle) | 1 | 1.00E+00 |
| ko03018 | RNA degradation | 1 | 1.00E+00 |
| ko03008 | Ribosome biogenesis in eukaryotes | 1 | 1.00E+00 |
| ko04144 | Endocytosis | 1 | 1.00E+00 |
| ko00510 | N-Glycan biosynthesis | 1 | 1.00E+00 |
| ko00240 | Pyrimidine metabolism | 1 | 1.00E+00 |
| ko03022 | Basal transcription factors | 1 | 1.00E+00 |
| ko04141 | Protein processing in endoplasmic reticulum | 1 | 1.00E+00 |
| ko03013 | RNA transport | 1 | 1.00E+00 |
| ko03040 | Spliceosome | 1 | 1.00E+00 |
