# Supplementary material for: De Novo Transcriptome Assembly and Characterization of the Synthesis Genes of Bioactive Constituents in Abelmoschus esculentus (L.) Moench
Source: Genes (Basel). 2018 Feb 27;9(3):130. doi: 10.3390/genes9030130 (PMC5867851; doi:10.3390/genes9030130)
Supplement: Supplementary file 1 [file genes-09-00130-s001.zip › Supplemental final/Table S5.docx]

**T**able S5: The information of 30,415 DEGs in “Fl vs Fr” comparison were assigned to 128 KEGG pathways

| **Pathway ID** | **Pathway** | **Pvalue** | **Qvalue** |
| --- | --- | --- | --- |
| ko03010 | Ribosome | 0 | 0.00E+00 |
| ko01110 | Biosynthesis of secondary metabolites | 4.79E-32 | 3.07E-30 |
| ko01100 | Metabolic pathways | 1.81E-25 | 7.72E-24 |
| ko00500 | Starch and sucrose metabolism | 6.73E-24 | 2.15E-22 |
| ko00511 | Other glycan degradation | 8.66E-22 | 2.22E-20 |
| ko00270 | Cysteine and methionine metabolism | 2.93E-21 | 6.25E-20 |
| ko04075 | Plant hormone signal transduction | 1.44E-18 | 2.63E-17 |
| ko00195 | Photosynthesis | 3.28E-16 | 5.25E-15 |
| ko00940 | Phenylpropanoid biosynthesis | 1.49E-15 | 2.12E-14 |
| ko00604 | Glycosphingolipid biosynthesis - ganglio series | 8.35E-15 | 1.07E-13 |
| ko00410 | beta-Alanine metabolism | 2.28E-14 | 2.65E-13 |
| ko00061 | Fatty acid biosynthesis | 4.70E-13 | 5.02E-12 |
| ko00130 | Ubiquinone and other terpenoid-quinone biosynthesis | 7.48E-13 | 7.36E-12 |
| ko00330 | Arginine and proline metabolism | 1.38E-12 | 1.26E-11 |
| ko00040 | Pentose and glucuronate interconversions | 1.59E-12 | 1.35E-11 |
| ko00710 | Carbon fixation in photosynthetic organisms | 6.64E-12 | 5.31E-11 |
| ko00196 | Photosynthesis - antenna proteins | 1.22E-10 | 9.17E-10 |
| ko00520 | Amino sugar and nucleotide sugar metabolism | 2.50E-10 | 1.78E-09 |
| ko00910 | Nitrogen metabolism | 1.59E-09 | 1.07E-08 |
| ko00460 | Cyanoamino acid metabolism | 1.62E-08 | 1.04E-07 |
| ko00620 | Pyruvate metabolism | 7.75E-08 | 4.72E-07 |
| ko00260 | Glycine, serine and threonine metabolism | 1.38E-07 | 8.01E-07 |
| ko00053 | Ascorbate and aldarate metabolism | 2.17E-07 | 1.21E-06 |
| ko00603 | Glycosphingolipid biosynthesis - globo series | 2.45E-07 | 1.31E-06 |
| ko00250 | Alanine, aspartate and glutamate metabolism | 7.28E-07 | 3.73E-06 |
| ko00860 | Porphyrin and chlorophyll metabolism | 9.54E-07 | 4.70E-06 |
| ko00531 | Glycosaminoglycan degradation | 2.10E-06 | 9.96E-06 |
| ko00750 | Vitamin B6 metabolism | 9.90E-06 | 4.53E-05 |
| ko00600 | Sphingolipid metabolism | 1.44E-05 | 6.36E-05 |
| ko00290 | Valine, leucine and isoleucine biosynthesis | 1.92E-05 | 8.20E-05 |
| ko00071 | Fatty acid metabolism | 2.02E-05 | 8.33E-05 |
| ko00300 | Lysine biosynthesis | 2.84E-05 | 1.14E-04 |
| ko00670 | One carbon pool by folate | 0.000153532 | 5.96E-04 |
| ko00360 | Phenylalanine metabolism | 0.00024253 | 8.91E-04 |
| ko00052 | Galactose metabolism | 0.000243666 | 8.91E-04 |
| ko00909 | Sesquiterpenoid and triterpenoid biosynthesis | 0.000316974 | 1.13E-03 |
| ko00010 | Glycolysis / Gluconeogenesis | 0.000362682 | 1.25E-03 |
| ko00770 | Pantothenate and CoA biosynthesis | 0.000540407 | 1.76E-03 |
| ko00051 | Fructose and mannose metabolism | 0.000547136 | 1.76E-03 |
| ko00944 | Flavone and flavonol biosynthesis | 0.000550225 | 1.76E-03 |
| ko00906 | Carotenoid biosynthesis | 0.001071914 | 3.35E-03 |
| ko00970 | Aminoacyl-tRNA biosynthesis | 0.001326351 | 4.04E-03 |
| ko00630 | Glyoxylate and dicarboxylate metabolism | 0.002262705 | 6.74E-03 |
| ko00945 | Stilbenoid, diarylheptanoid and gingerol biosynthesis | 0.003101847 | 9.02E-03 |
| ko00380 | Tryptophan metabolism | 0.003803623 | 1.08E-02 |
| ko00100 | Steroid biosynthesis | 0.005849316 | 1.59E-02 |
| ko00480 | Glutathione metabolism | 0.005850241 | 1.59E-02 |
| ko00340 | Histidine metabolism | 0.006655119 | 1.77E-02 |
| ko00941 | Flavonoid biosynthesis | 0.006969173 | 1.82E-02 |
| ko00660 | C5-Branched dibasic acid metabolism | 0.009457423 | 2.42E-02 |
| ko00780 | Biotin metabolism | 0.01220615 | 3.06E-02 |
| ko00740 | Riboflavin metabolism | 0.01614329 | 3.97E-02 |
| ko03030 | DNA replication | 0.02013329 | 4.86E-02 |
| ko00592 | alpha-Linolenic acid metabolism | 0.0208955 | 4.95E-02 |
| ko03410 | Base excision repair | 0.04474083 | 1.04E-01 |
| ko00942 | Anthocyanin biosynthesis | 0.06216863 | 1.41E-01 |
| ko03008 | Ribosome biogenesis in eukaryotes | 0.06297007 | 1.41E-01 |
| ko00903 | Limonene and pinene degradation | 0.06428181 | 1.42E-01 |
| ko00280 | Valine, leucine and isoleucine degradation | 0.07362571 | 1.60E-01 |
| ko00350 | Tyrosine metabolism | 0.08285865 | 1.77E-01 |
| ko00073 | Cutin, suberine and wax biosynthesis | 0.1227056 | 2.57E-01 |
| ko04140 | Regulation of autophagy | 0.1348988 | 2.79E-01 |
| ko00400 | Phenylalanine, tyrosine and tryptophan biosynthesis | 0.166351 | 3.38E-01 |
| ko00402 | Benzoxazinoid biosynthesis | 0.1817745 | 3.64E-01 |
| ko00232 | Caffeine metabolism | 0.1984921 | 3.91E-01 |
| ko00966 | Glucosinolate biosynthesis | 0.2043504 | 3.91E-01 |
| ko01040 | Biosynthesis of unsaturated fatty acids | 0.2044566 | 3.91E-01 |
| ko00790 | Folate biosynthesis | 0.2112219 | 3.98E-01 |
| ko03430 | Mismatch repair | 0.2237334 | 4.15E-01 |
| ko00640 | Propanoate metabolism | 0.3066843 | 5.61E-01 |
| ko03440 | Homologous recombination | 0.4840706 | 8.73E-01 |
| ko00950 | Isoquinoline alkaloid biosynthesis | 0.5437845 | 9.54E-01 |
| ko03450 | Non-homologous end-joining | 0.5438845 | 9.54E-01 |
| ko00020 | Citrate cycle (TCA cycle) | 0.567266 | 9.69E-01 |
| ko00960 | Tropane, piperidine and pyridine alkaloid biosynthesis | 0.5679811 | 9.69E-01 |
| ko00591 | Linoleic acid metabolism | 0.583893 | 9.83E-01 |
| ko00590 | Arachidonic acid metabolism | 0.6012173 | 9.94E-01 |
| ko00565 | Ether lipid metabolism | 0.6055741 | 9.94E-01 |
| ko00310 | Lysine degradation | 0.6411841 | 1.00E+00 |
| ko00563 | Glycosylphosphatidylinositol(GPI)-anchor biosynthesis | 0.6826662 | 1.00E+00 |
| ko00943 | Isoflavonoid biosynthesis | 0.6827933 | 1.00E+00 |
| ko00730 | Thiamine metabolism | 0.6964959 | 1.00E+00 |
| ko00062 | Fatty acid elongation | 0.7723225 | 1.00E+00 |
| ko00901 | Indole alkaloid biosynthesis | 0.7799403 | 1.00E+00 |
| ko00450 | Selenocompound metabolism | 0.8690733 | 1.00E+00 |
| ko00902 | Monoterpenoid biosynthesis | 0.8810172 | 1.00E+00 |
| ko00072 | Synthesis and degradation of ketone bodies | 0.8810172 | 1.00E+00 |
| ko04145 | Phagosome | 0.9103273 | 1.00E+00 |
| ko03013 | RNA transport | 0.9253699 | 1.00E+00 |
| ko00430 | Taurine and hypotaurine metabolism | 0.940839 | 1.00E+00 |
| ko00650 | Butanoate metabolism | 0.9436931 | 1.00E+00 |
| ko04122 | Sulfur relay system | 0.9681971 | 1.00E+00 |
| ko04710 | Circadian rhythm - mammal | 0.9820656 | 1.00E+00 |
| ko04146 | Peroxisome | 0.9865745 | 1.00E+00 |
| ko00904 | Diterpenoid biosynthesis | 0.9875096 | 1.00E+00 |
| ko00510 | N-Glycan biosynthesis | 0.9877503 | 1.00E+00 |
| ko00908 | Zeatin biosynthesis | 0.9881571 | 1.00E+00 |
| ko00785 | Lipoic acid metabolism | 0.9912011 | 1.00E+00 |
| ko00965 | Betalain biosynthesis | 0.9948416 | 1.00E+00 |
| ko00760 | Nicotinate and nicotinamide metabolism | 0.9949126 | 1.00E+00 |
| ko00514 | Other types of O-glycan biosynthesis | 0.9958405 | 1.00E+00 |
| ko00905 | Brassinosteroid biosynthesis | 0.9980765 | 1.00E+00 |
| ko00230 | Purine metabolism | 0.9981387 | 1.00E+00 |
| ko00030 | Pentose phosphate pathway | 0.9985769 | 1.00E+00 |
| ko00561 | Glycerolipid metabolism | 0.999295 | 1.00E+00 |
| ko00920 | Sulfur metabolism | 0.9993144 | 1.00E+00 |
| ko00900 | Terpenoid backbone biosynthesis | 0.9993352 | 1.00E+00 |
| ko04144 | Endocytosis | 0.9997049 | 1.00E+00 |
| ko00564 | Glycerophospholipid metabolism | 0.999845 | 1.00E+00 |
| ko04650 | Natural killer cell mediated cytotoxicity | 0.9998965 | 1.00E+00 |
| ko04712 | Circadian rhythm - plant | 0.9999063 | 1.00E+00 |
| ko04120 | Ubiquitin mediated proteolysis | 0.9999182 | 1.00E+00 |
| ko03420 | Nucleotide excision repair | 0.9999814 | 1.00E+00 |
| ko03020 | RNA polymerase | 0.9999995 | 1.00E+00 |
| ko03022 | Basal transcription factors | 1 | 1.00E+00 |
| ko03050 | Proteasome | 1 | 1.00E+00 |
| ko03060 | Protein export | 1 | 1.00E+00 |
| ko04070 | Phosphatidylinositol signaling system | 1 | 1.00E+00 |
| ko00240 | Pyrimidine metabolism | 1 | 1.00E+00 |
| ko04130 | SNARE interactions in vesicular transport | 1 | 1.00E+00 |
| ko04141 | Protein processing in endoplasmic reticulum | 1 | 1.00E+00 |
| ko04626 | Plant-pathogen interaction | 1 | 1.00E+00 |
| ko02010 | ABC transporters | 1 | 1.00E+00 |
| ko03040 | Spliceosome | 1 | 1.00E+00 |
| ko03015 | mRNA surveillance pathway | 1 | 1.00E+00 |
| ko03018 | RNA degradation | 1 | 1.00E+00 |
| ko00190 | Oxidative phosphorylation | 1 | 1.00E+00 |
| ko00562 | Inositol phosphate metabolism | 1 | 1.00E+00 |
